# Evaluation of traditional Chinese exercise for knee osteoarthritis (KOA): an overview of systematic reviews

**DOI:** 10.1186/s13643-024-02606-0

**Published:** 2024-07-18

**Authors:** Tao Tao, Ming-peng Shi, Bo-yang Tan, Xian-shuai Zhang, Feng-ling Sun, Bao-ren Liu, Shao-jun Li, Zhen-hua Li

**Affiliations:** 1 College of Integrated Chinese and Western Medicine, Changchun University of Chinese Medicine, Changchun, 130117 China; 2 College of Traditional Chinese Medicine, Changchun University of Chinese Medicine, Changchun, 130117 China; 3 Affiliated Hospital of the Changchun University of Chinese Medicine, Changchun, 130021 China; 4 Taojiatun Town Health Center, Gongzhuling City, Changchun, 136104 China

**Keywords:** Knee osteoarthritis, Traditional Chinese exercise, AMSTAR-2, RPISMA 2020, GRADE, Overview of systematic reviews

## Abstract

**Background:**

Knee osteoarthritis (KOA) has become a public health issue. Several systematic reviews (SRs) and meta-analyses (MAs) indicate that traditional Chinese exercise (TCE) may be an effective treatment for reducing pain and stiffness and improving physical function in people with knee osteoarthritis (KOA).

**Objectives:**

To evaluate the literature quality and evidence for the systematic reviews of TCE for KOA and provide evidence to support the clinical application of TCE for KOA.

**Methods:**

Eight databases were searched from their inception to January 3, 2023, to retrieve relevant literature, including China National Knowledge Infrastructure (CNKI), Wanfang, Chinese Scientific Journal Database (VIP), China Biology Medical literature database (CBM), PubMed, Embase, Web of Science and Cochrane Library, without restrictions on publication date or language. AMSTAR-2 and PRISMA 2020 assessed the methodological and reporting quality of included SRs/MAs. The Grading of Recommendations Assessment, Development, and Evaluation (GRADE) system was utilized to evaluate the quality of evidence.

**Results:**

A total of 18 SRs/MAs were included. The methodological quality was “very low” based on AMSTAR-2. The overall reporting quality was deficient based on PRISMA 2020. The quality of Chinese and English literature differed, with English literature being superior in methodological and reporting quality. Among 93 pieces of evidence obtained, 46 (49.46%) were of very low quality, 34 (36.56%) were of low quality, 13 (13.98%) were of moderate quality, and none were of high quality. TCE was supported by 76 pieces of evidence (81.72%).

**Conclusion:**

TCE appears beneficial and safe for managing KOA. However, due to the relatively low methodological and evidentiary quality of included SRs/MAs, clinicians should interpret these findings cautiously.

**Supplementary Information:**

The online version contains supplementary material available at 10.1186/s13643-024-02606-0.

## Introduction

Knee osteoarthritis (KOA) is a degenerative disease of the joint cartilage, with subchondral bone lesions and synovial inflammation as the main manifestations [1]. The clinical symptoms include pain, joint stiffness, and functional impairment. The prevalence of symptomatic KOA in China is 8.1%, with a higher proportion in women than men and significant geographical differences [2]. In traditional Chinese Medicine (TCM), the pathological basis of KOA is a deficiency of the liver and kidney and invasion of wind, cold, and dampness. The knee joint becomes enlarged, flexion and extension become difficult, and mobility is restricted. The main pathological changes include articular cartilage damage, subchondral bone hardening or cystic changes, osteophyte formation at joint margins, apparent synovial lesions, joint capsule contracture, ligament loosening or contracture, muscle atrophy, and weakness [3]. This disease mainly occurs in middle-aged and elderly patients and belongs to the categories of “paralysis,” “bone paralysis,” “tendon paralysis,” “bone impotence,” and “tendon impotence” in TCM. The clinical manifestations include morning stiffness, unstable walking, pain, and functional impairment.

Pharmacotherapy, physical therapy, rehabilitation therapy, acupuncture, massage, surgery (including total knee replacement), and additional methods are all viable options for managing KOA. Nevertheless, the extended utilization of these medications presents a potential for a multitude of detrimental effects, including hypertension, renal toxicity, gastrointestinal impairment, congestive heart failure, and cardiovascular incidents [4]. Additionally, physical therapy is not appropriate for terminal patients who require surgical intervention, among other limitations. Early-stage KOA patients are treated nonoperatively; surgery is not needed [5]. It is critical to identify a viable nonsurgical intervention that can effectively mitigate symptoms in patients diagnosed with KOA, as early-stage surgery is not advised. A systematic review of therapeutic exercise for KOA suggests that patients may observe significant improvements in their physiological function, overall quality of life, and joint pain reduction [6].

The holistic concept of TCM considers the unity of body and spirit, and only when harmony between them can an organism maintain vitality and vigor. The separation of body and spirit indicates the end of life. Traditional Chinese exercise (TCE) is guided by the holistic concept of TCM, the theory of five elements and yin-yang, and the view of meridians and zang-fu organs [1, 7]. It has gradually formed a unique system that combines movement and stillness, dredges meridians, regulates qi and blood, focuses on strengthening the body, nourishing and controlling, and enhances the body to prevent diseases by combining ancient Chinese philosophy. Studies have shown Taijiquan effectively treats KOA [8], improves mental health, increases life satisfaction, and promotes [9, 10]. In addition to physical exercise, TCE pays more attention to psychological and spiritual adjustment, as well as the intervention of emotions and spirit, appropriate work and rest, and other factors that influence disease development to enhance the body’s defense ability, smooth the flow of qi and blood, harmonize zang-fu organs, and improve organ function, thus playing a role in disease prevention and treatment [11]. The commonly used TCE methods include Taiji, Yijinjing, Baduanjin, Wuqinxi, and Qigong.

The number of systematic reviews (SRs) and meta-analyses (MAs) on TCE interventions for KOA has been increasing in recent years. However, the quality of the literature and evidence needs to be determined. This study used AMSTAR-2 and PRISMA 2020 to assess the methodological and reporting quality and GRADE to evaluate the evidence quality to objectively reflect the current status of the evidence-based evaluation of TCE. The aims were to systematically and critically assess SRs/MAs of TCE interventions for KOA and provide a reference for future evaluation studies of TCE and the development of evidence-based guidelines.

## Materials and methods

### Design and registration

All analyses were based on previously published data. Therefore, no ethical approval or patient consent was required. The methodology of the overview of systematic reviews (SRs) followed the Cochrane Handbook [12] and the Preferred Reporting Items for Systematic Reviews and Meta-Analyses (PRISMA) guideline. This study was also conducted and reported under the guidance of the checklist of Preferred Reporting Items for the Overview of Systematic Reviews (PRIO-harms) [13].

### Eligibility criteria

#### Inclusion criteria

1. Types of studies

This study type is a thorough Systematic Review and Meta-analyses (SRs/Mas) of TCE for knee osteoarthritis based on a randomized controlled trial in any language; the randomized controlled trial is the gold standard for evaluating treatments. In clinical research, the random assignment approach is essential. According to this procedure, each individual has an equal chance of being assigned to the experimental or control group, which employs randomization.

(2). Types of participants

The study population is anyone who meets the American College of Rheumatology diagnostic standards, the Chinese Medical Association Orthopedic Branch, or the domestic industry norm for Western or Chinese Medicine KOA, regardless of gender, age, or location.

(3). Types of interventions

 

Interventions: Traditional Chinese exercise methods, such as Taiji, Baduanjin, Wuqinxi, Yijinjing, Qigong, etc., are used in the treatment group; the control group may consist of conventional exercise, conventional care, health education, or blank control, as well as additional therapies that are not part of the test group.

(4). Types of outcomes

The final index should have at least one different index for each: pain, stiffness, physiological function score, quality of life, safety, etc.

#### Exclusion criteria

(1) Duplicate publications; (2) reviews, animal studies, case reports, conference papers, abstracts, books, comments; (3) mixed hip osteoarthritis in study participants; (4) co-interventions of other complementary and alternative therapies in addition to TCE methods (e.g., massage, acupuncture, herbal therapy, moxibustion, transcutaneous electrical nerve stimulation, cupping, gua sha, bath therapy); (5) protocols for SRs/MAs; (6) Network meta-analyses; (7) insufficient data information for data extraction; (8) full text was not available.

### Search strategy

The following eight databases were searched from their inception to January 3, 2023: China National Knowledge Infrastructure (CNKI), Wanfang, Chinese Scientific Journal Database (VIP), China Biology Medical Literature Database (CBM), PubMed, Embase, Web of Science and Cochrane Library, without restrictions on publication date or language. Reference lists of included studies were also reviewed to identify any relevant papers missed in the search. Additionally, trial registries, relevant grey literature, and consultation with experts in related fields were manually searched. Two reviewers conducted the literature search independently. The search terms included Taiji, Tai Chi, Tai Ji, Taichi, T'ai Ji, T'ai Chi, Taichiquan, Taijiquan, T'ai Ji Quan, T'ai Chi Chuan, Baduanjin, Eight-Section Brocade, Yijinjing, Classic of Changing Tendon, Yi-Gin-Ching, Wuqinxi, Five Animals Exercise, Qigong, Traditional Chinese Exercise, Traditional Chinese Medicine Exercise, Remedial Exercise, Therapeutic Exercise; Osteoarthrit*, Knee Osteoarthritis, Gonarthritis, KOA, Osteoarthritis Knee, Degenerative Arthritis; Meta-Analysis, Meta-Analyses, Data Pooling, Systematic Review. The detailed search strategies in Web of Science databases are shown in Additional file 1: Appendix 1.

### Study selection and data extraction

#### Data source and eligibility

EndNote X9 software was utilized to screen the literature. Duplicates were removed using the software and manual examination. Titles and abstracts were read carefully, and those not meeting the inclusion criteria were excluded. The complete manuscripts of the remaining studies were downloaded and reviewed thoroughly, and studies not matching the inclusion criteria in interventions, outcomes, or participants were eliminated. Studies with insufficient information were also excluded. The final studies that were included were determined after discussion and analysis.

#### Data extraction

Two reviewers extracted data independently using a predesigned form according to the inclusion and exclusion criteria and cross-checked with each other. Any disagreements were resolved through discussion with a third reviewer making the final decision. The critical information of included studies was summarized in a table, including first author, year of publication, number of studies (articles), sample size (participants), interventions in treatment and control groups, risk of bias assessment tool, outcome indicators, and main findings.

### Methodological quality assessment

#### Methodological quality assessment

Two independent reviewers assessed the methodological quality of included SRs/MAs using the AMSTAR-2 tool [14]. Any disagreements were resolved through discussion with a third reviewer making the final decision. Each item was judged as “yes,” “partial yes,” or “no.” “Yes” means the study fully addressed and substantiated the issue raised in the item, while “partial yes” indicates only part of the issue was addressed. “No” means the study did not substantiate or incorrectly examine the issue raised in the item due to insufficient information or absence of data.

Based on the criticality of each item and the evaluation results, the methodological quality of each SR/MA was categorized as high, moderate, low, or critically low: high quality—no or only one non-critical weakness; moderate quality—more than one non-critical weakness; low quality—one critical flaw with or without non-critical deficiencies; critically low quality—more than one essential flaw with or without non-critical faults.

#### Assessment of reporting quality

Two independent reviewers assessed the reporting quality of SRs/MAs using the PRISMA 2020 checklist [15, 16]. Any disagreements were resolved through discussion with a third reviewer making the final decision. Each item was judged as “fully reported,” “partially reported,” or “not reported” based on compliance with the reporting requirements [17].

#### Assessment of the risk of bias 

Two independent reviewers assessed the risk of bias for SRs/MAs using the Risk of ROBIS tool [18]. The ROBIS tool aims to evaluate the level of bias presented in a systematic review. This bias assessment tool covers three phases: (1) assessing relevance (optional according to the situation); (2) identifying concerns with the review process (study eligibility criteria, identification and selection of studies, data collection and study appraisal, synthesis, and findings); (3) judging the risk of bias. The results were rated as “high risk,” “low risk,” or “unclear risk.”

#### Assessment of evidence quality

Two independent reviewers assessed the quality of clinical evidence for SRs/MAs using the GRADE approach [19–21]. Any disagreements were resolved through discussion with a third reviewer making the final decision. The main reasons for downgrading evidence quality include limitations in study design, inconsistency of results, indirectness of evidence, imprecision, and publication bias. In addition, if no downgrading was present, the quality of evidence was considered high, with one downgrade, moderate; with two downgrades, low; with three or more downgrades, very low.

### Data synthesis

A narrative synthesis approach was utilized. Outcome data were presented as per the original SRs/MAs, and no additional re-analysis of data was conducted. Data were extracted and plotted using WPS 2022 to generate tables. A descriptive analysis was performed to present the literature quality, evidence quality, and main findings of the included studies.

## Results

### Literature search and selection

**Fig. 1:**
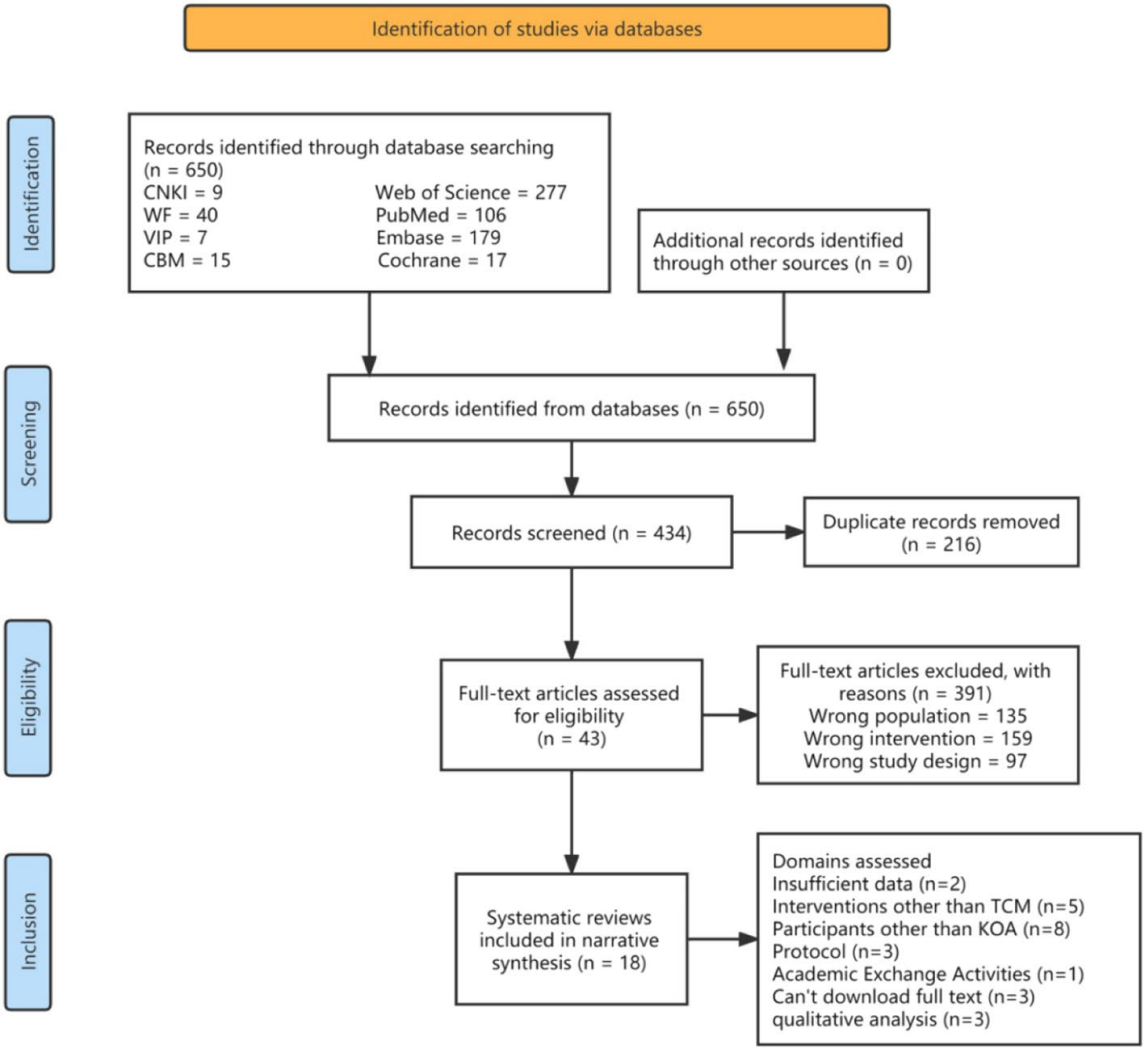
Flow chart of literature screening

The initial search yielded 650 records. After removing 216 duplicates using EndNote X9, 391 records remained. Screening titles and abstracts resulted in the exclusion of 241 articles. After reading the full text of the remaining 50 articles, 23 were further excluded, including three that were unavailable in full text, 1 abstract from an academic conference, 5 containing interventions other than TCM methods, 8 featuring participants without knee osteoarthritis, 3 protocols for SRs/MAs, and 2 with insufficient information. Another 3 articles were excluded as they only conducted qualitative analysis. Reviewing all included studies generated a summary of clinical efficacy evaluation methodologies and criteria systems for TCM interventions in knee osteoarthritis. Finally, 18 studies were included. A comprehensive review framework was established after collecting, organizing, analyzing, and synthesizing relevant literature. The screening process is outlined in Fig. 1.

### Characteristics of included reviews

A total of 18 SRs/Mas [22–39] were included, among which 10 [22–31] were in English and 8 [32–39] were in Chinese. Additionally, there were 17 journal articles [22–32, 34–39] and 1 dissertation [33]. The publication years have spanned from 2013 to 2022. Fifteen studies [22, 24–30, 32, 33, 35–39] focused on Taiji, 6 [27, 30–34] involved Baduanjin, 2 [32, 33] included Yijinjing, and 3 [23, 32, 33] examined Wuqinxi. Fifteen studies [23, 25–27, 29–39] utilized the Cochrane risk of bias tool, 2 [24, 28] used the Jadad scale, and 1 [22] employed AMSTAR 2. The primary characteristics of the included reviews are presented in Table 1.

**Table 1:** Basic characteristics of included reviews

Citation	N Primary studies	N of participants	Intervention group	Control group	Quality assessment	Outcomes	Main conclusion
George A Kelley [22] 2022	8	407	Tai Chi	Control group	AMSTAR 2	WOMAC pain, WOMAC stiffness, WOMAC physical function	Tai Chi results in statistically significant and clinically meaningful improvements in pain, stiffness, and physical function across a wide range of ACR in adults with KOA
Jiale Guo [23] 2022	7	668	Wuqinxi	Blank control, physical treatment	Cochrane	WOMAC, VAS	A definite effect of WQX in improving pain symptoms and joint function in patients with KOA
Jun-Hong Yan [24] 2013	7	348	Tai Chi	Any type of control	Jadad	WOMAC pain, WOMAC stiffness, WOMAC physical function	Twelve-week Tai Chi improves arthritic symptoms and physical function in patients with osteoarthritis and should be included in rehabilitation programs
Lidong Hu [25] 2021	16	986	Tai Chi	No exercise, education class, standard care, or physical therapy	Cochrane	WOMAC pain, WOMAC stiffness, WOMAC physical function, 6 MWT, TUG, balance score, SF-36 PCS, SF-36 MCS, depression score, ASES	Tai Chi exercise alleviates the physical and mental health of patients with knee osteoarthritis and should be available as an alternative non-pharmacological therapy in rehabilitation programs
R. Lauche [26] 2013	5	252	Tai Chi	No treatment, usual care, placebo, or any active treatment	Cochrane	WOMAC/VAS pain, WOMAC stiffness, WOMAC physical function, SF-36 PCS, SF-36 MCS	This systematic review found moderate evidence for short-term improvement of pain, physical function, and stiffness in patients with osteoarthritis of the knee practicing Tai Chi
Ruojin Li [27] 2020	14	815	Tai Chi /Baduanjin	Physical therapy, health education, and sham exercise	Cochrane	WOMAC/KOOS pain, WOMAC/KOOS- stiffness, WOMAC/KOOS physical function	TCE may be effective in alleviating pain, relieving stiffness, and improving the physical position of patients with KO
Wen-Dien Chang [28] 2016	11	508	Tai Chi	Education class, telephone interviews	Jadad	WOMAC pain, WOMAC stiffness, WOMAC physical function, 6 MWT, stair climb test, SAFE	Tai Chi Chuan had beneficial outcomes for patients with knee osteoarthritis. The evidence-based results represented that it had small-to-moderate effects on body functions and structures, activities, and participation of physical components
Yanwei You [29] 2021	11	603	Tai Chi	Attention control	Cochrane	6 MWT, TUG, WOMAC physical function	Tai Chi could be an excellent physical training strategy for improving walking function and posture control in older adults with knee osteoarthritis
Yingjie Zhang [30] 2017	8	375	Tai Chi/Baduanjin	Receiving wellness education or no treatment	Cochrane	WOMAC/VAS pain, WOMAC stiffness, WOMAC physical function, SF-36 PCS, SF-36 MCS	Our systematic review revealed that short-term TCE could reduce pain, improve physical function, and alleviate stiffness. These results suggest that TCE could be helpful as an adjuvant treatment for patients with knee OA
Zhi-peng Zeng [31] 2020	7	424	Baduanjin	Waiting list, placebo control, NSAID therapy, and health education	Cochrane	WOMAC pain, WOMAC stiffness, WOMAC physical function	Baduanjin exercise may have favorable effects on KOA patients
ZENG Ling-feng [32] 2018	11	670	Tai Chi/Wuqinxi/Baduanjin/Yijinjing	Control group	Cochrane	WOMAC pain, WOMAC stiffness, WOMAC physical function, SF-36	Traditional exercise Taijiquan has a remarkable efficacy on knee osteoarthritis and relatively fewer adverse reactions and has positive effects on improving pain and joint function in patients with knee osteoarthritis
Li Ruojin [33] 2021	28	1800	Tai Chi/Wuqinxi /Baduanjin /Yijinjing	Control group	Cochrane	WOMAC/KOOS pain, WOMAC/KOOS stiffness, WOMAC/KOOS physical function	Traditional Chinese exercise can effectively relieve joint pain and improve common stiffness symptoms and physical function in patients with knee osteoarthritis, and it has particular safety
Li Zimeng [34] 2020	6	328	Baduanjin	Blank control, usual care, health education, and usual exercise	Cochrane	WOMAC/VAS pain, WOMAC stiffness, WOMAC physical function, 6 MWT	Baduanjin has significant clinical effects on knee osteoarthritis patients’ pain, stiffness, and mobility
WANG Li-dong [35] 2022	16	917	Tai Chi	No exercise, usual care, and Conservative treatments	Cochrane	WOMAC/KOOS pain, stiffness, physical function, 6 MWT, TUG	Taijiquan exercise can improve pain, function, stiffness, and sports performance in KOA patients
LIU Wen-jun [36] 2020	15	734	Tai Chi	Health education, Routine nursing, meditation	Cochrane	WOMAC pain, WOMAC stiffness, WOMAC physical function, TUG, 6 MWT	Tai chi exercises significantly improve joint pain, stiffness, overall dysfunction, and walking speed in people with osteoarthritis while maintaining a high level of safety
XIA Lu-qin [37] 2020	8	374	Tai Chi	Blank control, health education	Cochrane	WOMAC	Tai chi exercise for more than 160 min per week (about 3 h) for 16 weeks can successfully alleviate the symptoms of difficulties performing daily activities in patients with knee osteoarthritis. It can play an essential role in treating knee osteoarthritis in the elderly
XIE Hui [38] 2016	12	542	Tai Chi	Blank control, usual care, health education, and usual exercise	Cochrane	WOMAC/ASES pain, WOMAC/ASES physical function. WOMAC stiffness, TUG, STS, 6MWT, balance score, SF-12/36	Taijiquan could improve pain and joint function in patients with OA effectively without any significant adverse effects
XIE Yu [39] 2015	7	367	Tai Chi	Blank control, telephone interviews, health education, placebo	Cochrane	Pain/physical function/stiffness, balance score, SF-36 PCS, SF-36 MCS, 6 MWT, BMI, Muscle strength (extensor/flexor)	Tai Chi benefits knee osteoarthritis patients’ joint pain, joint stiffness, joint function, walking speed, and somatic quality of life while being safe

*WOMAC* Western Ontario and McMaster Universities Arthritis Index, *VAS* visual analog scale, *STS* sit-to-stand test, *TUG* time up and test, *6 MWT* 6-min walk test, *SAFE* Survey of Activities and Fear of Falling in the Elderly, *KOOS* Knee Injury, and Osteoarthritis Outcome Score, *SF-12* Short Form Health Survey, *SF-36* 36-Item Short Form Health Survey, *UPST* uni pedal stance test, *ASES* arthritis self-efficacy scale, *BMI* body mass index

### Methodological and reporting quality assessment

#### Methodological quality assessment

The AMSTAR-2 evaluation outcomes are shown in Table 2. The methodological quality of all 18 included SRs was rated as “critically low.” Based on analysis of the 7 critical items, the main flaws were the following: (1) item 2: only 16.67% (3/18) studies provided registration information, while the remaining lacked registration and protocol-related contents; (2) item 4: although 100% (18/18) studies searched ≥ 2 databases, only 11.11% (2/18) performed additional searches such as checking reference lists or grey literature; (3) item 7: none of the 18 studies mentioned excluded studies; (4) item 9: only 11.11% (2/18) studies assessed risk of bias from randomization and blinding, and selective outcome reporting; (5) item 11: 55.56% (10/18) studies did not use appropriate methods for conducting meta-analysis; (6) item 13: 27.78% (5/18) studies did not examine the potential impact of risk of bias in included studies on the effect estimate; (7) Item 15: among 18 studies, 44.44% (8/18) did not assess for publication bias using funnel plots or statistical tests like Egger’s test. Based on the above assessment, all the included reviews were rated as “critically low” in methodological quality.

**Table 2:** The evaluation results of methodological quality based on AMSTAR-2

Authors, year	Q 1	Q 2^*^	Q 3	Q 4^*^	Q 5	Q 6	Q 7^*^	Q 8	Q 9^*^	Q 10	Q 11^*^	Q 12	Q 13^*^	Q 14	Q 15^*^	Q 16	Overall quality
George A Kelley 2022 [22]	Y	Y	N	Y	Y	Y	N	N	N	N	Y	N	Y	Y	N	Y	VL
Jiale Guo 2022 [23]	Y	Y	N	PY	Y	Y	N	Y	Y	N	Y	N	N	Y	N	Y	VL
Jun-Hong Yan 2013 [24]	Y	N	N	PY	Y	Y	N	PY	Y	N	N	Y	N	Y	Y	N	VL
Lidong Hu 2021 [25]	Y	N	N	PY	Y	Y	N	Y	Y	N	N	N	Y	N	Y	Y	VL
R. Lauche 2013 [26]	Y	N	N	PY	Y	Y	N	Y	Y	N	N	Y	Y	N	Y	Y	VL
Ruojin Li 2020 [27]	Y	N	N	PY	Y	Y	N	PY	Y	N	N	N	Y	N	Y	Y	VL
Wen-Dien Chang 2016 [28]	N	N	N	PY	N	N	N	N	Y	N	N	N	Y	N	N	Y	VL
Yanwei You 2021 [29]	Y	N	N	PY	N	Y	N	PY	Y	N	Y	N	N	Y	N	N	VL
Yingjie Zhang 2017 [30]	Y	N	N	PY	Y	Y	N	Y	Y	N	N	N	Y	Y	N	N	VL
Zhi-peng Zeng 2020 [31]	Y	Y	N	Y	Y	Y	N	PY	Y	N	Y	Y	Y	Y	N	Y	VL
ZENG Ling-feng 2018 [32]	Y	N	N	PY	Y	N	N	PY	Y	N	N	N	Y	N	Y	N	VL
Li Ruojin 2021 [33]	Y	N	N	PY	N	N	N	PY	Y	N	Y	Y	Y	Y	Y	N	VL
Li Zimeng 2020 [34]	Y	N	N	PY	N	Y	N	PY	Y	N	N	N	Y	Y	N	N	VL
WANG Li-dong 2022 [35]	Y	N	N	PY	Y	Y	N	PY	Y	N	Y	Y	N	Y	Y	N	VL
LIU Wen-jun 2020 [36]	Y	N	N	PY	N	N	N	PY	Y	N	N	Y	Y	N	Y	N	VL
XIA Lu-qin 2020 [37]	Y	N	N	PY	N	N	N	N	N	N	Y	N	N	Y	N	N	VL
XIE Hui 2016 [38]	Y	N	N	PY	Y	Y	N	PY	Y	N	N	Y	Y	N	Y	N	VL
XIE Yu 2015 [39]	Y	N	N	PY	Y	Y	N	PY	Y	N	Y	N	Y	Y	Y	N	VL

*Y* yes, *PY* partial yes, *N* no, *VL* very low, *L* low, *M* medium, *H* high

*Key items that affect the quality of systematic evaluation methodology

Further analysis revealed that the average number of fully addressed items was 7.5 for English literature versus 6 for Chinese literature; the average number of non-addressed items was 7.3 in English literature compared to 8.125 in Chinese literature. This indicates differences between English and Chinese literature regarding the number of “yes” and “no” items.

#### Report quality of the included SRs/MAs

RPISMA 2020 was used to evaluate the reporting quality of the included studies. The results are presented in Table 3. The key reporting flaws are 77.78% (14/18) of the studies identified themselves as a systematic review; 83.33% (15/18) partially reported the Abstracts checklist; 94.44% (17/18) did not report using supplementary search techniques; 77.78% (14/18) provided incomplete search strategies, with only 11.11% (2/18) giving search strategies for all databases in the appendix; 100% (18/18) did not report funding sources; 100% (18/18) did not explain preprocessing (e.g., handling of missing summary statistics or data conversions) before data merger; 33.33% (6/18) did not discuss strategies to examine heterogeneity (e.g., subgroup analysis, meta-regression); 50%(9/18) did not mention methods to assess results stability (e.g., sensitivity analysis); 50% (9/18) did not evaluate inclusion of studies with publication bias; 83.33% (15/18) did not use the GRADE system to rate the quality of evidence; 38.89% (7/18) did not provide results of risk of bias assessment; 33.33% (6/18) did not describe the characteristics of any composite outcome and the potential for bias across studies; 16.67% (3/18) did not provide full statistically-based results; 50% (9/18) did not present findings of investigation into potential sources of study heterogeneity; 50% (9/18) lacked sensitivity analysis results; 44.44% (8/18) did not provide risk assessment of bias due to missing data (reporting bias); 83.33% (15/18) did not provide any supporting documentation for their grade; 16.67% (3/18) did not analyze findings using additional data; 11.11% (2/18) of included studies in the systematic review did not discuss their limitations; 16.67% (3/18) did not discuss limitations of the review process; 83.33% (15/18) did not mention registration or stated they were not registered; 83.33% (15/18) did not provide access to a protocol or expressed there was none; 55.56% (10/18) did not describe funding source or the funder’s role; 55.56% (10/18) authors conducting the systematic reviews did not declare any conflicts of interest; 66.67% (12/18) data came from sources not publicly available (e.g., data extraction form templates).

**Table 3:** The evaluation results of reporting quality based on PRISMA 2020

Items	George A Kelley 2022	Jiale Guo 2022	Jun-Hong Yan 2013	Lidong Hu 2021	R. Lauche 2013	Ruojin Li 2020	Wen-Dien Chang 2016	Yanwei You 2021	Yingjie Zhang 2017	Zhi-peng Zeng 2020	ZENG Ling-feng 2018	Li Ruojin 2021	Li Zimeng 2020	WANG Li-dong 2022	LIU Wen-jun 2020	XIA Lu-qin 2020	XIE Hui 2016	XIE Yu 2015	Number of yes or partially yes (%)
Items1	Y	Y	N	Y	Y	Y	Y	Y	Y	Y	Y	Y	N	Y	N	N	Y	Y	77.78%
Items2	N	Y	PY	PY	PY	PY	N	PY	PY	PY	PY	PY	PY	PY	PY	PY	PY	PY	88.89%
Items3	Y	Y	Y	Y	Y	Y	Y	Y	Y	Y	Y	Y	Y	Y	Y	N	Y	Y	94.44%
Items4	Y	Y	Y	Y	Y	Y	Y	Y	Y	Y	Y	Y	Y	Y	Y	Y	Y	Y	100%
Items5	N	PY	PY	PY	PY	PY	N	PY	PY	PY	PY	PY	PY	PY	PY	PY	PY	PY	88.89%
Items6	PY	PY	PY	PY	PY	PY	PY	PY	PY	Y	PY	PY	PY	PY	PY	PY	PY	PY	100%
Items7	Y	PY	PY	N	PY	PY	N	PY	PY	PY	PY	PY	PY	PY	PY	Y	PY	PY	88.89%
Items8	Y	Y	Y	Y	Y	Y	N	N	Y	Y	Y	N	N	Y	N	N	Y	Y	66.67%
Items9	Y	Y	Y	Y	Y	Y	N	Y	Y	Y	N	N	Y	Y	N	N	Y	Y	72.22%
Items10a	Y	Y	Y	Y	Y	Y	N	Y	Y	Y	Y	Y	Y	Y	Y	N	Y	Y	88.89%
Items10b	PY	PY	PY	PY	PY	PY	PY	PY	PY	PY	PY	PY	PY	PY	PY	N	PY	PY	94.44%
Items11	Y	Y	Y	Y	Y	Y	Y	Y	Y	Y	Y	Y	Y	Y	N	Y	Y	Y	94.44%
Items12	Y	Y	Y	Y	Y	Y	Y	Y	Y	Y	Y	Y	Y	Y	Y	Y	Y	Y	100%
Items13a	N	Y	Y	Y	Y	Y	N	N	Y	Y	Y	Y	Y	Y	Y	N	Y	Y	77.78%
Items13b	N	N	N	N	N	N	N	N	N	N	N	N	N	N	N	N	N	N	0%
Items13c	Y	Y	N	N	N	N	N	N	N	N	N	N	N	Y	N	N	N	N	16.67%
Items13d	Y	Y	Y	N	Y	Y	N	Y	Y	Y	Y	Y	Y	Y	N	Y	Y	Y	83.33%
Items13e	N	Y	Y	N	Y	Y	Y	N	N	Y	Y	Y	Y	Y	N	Y	Y	N	66.67%
Items13f	N	Y	Y	N	Y	Y	N	N	Y	Y	N	Y	Y	N	N	N	Y	N	50%
Items14	N	N	Y	Y	Y	Y	N	N	N	Y	Y	Y	N	N	N	N	Y	Y	50%
Items15	N	N	N	Y	N	N	N	N	N	N	Y	N	Y	N	N	N	N	N	16.67%
Items16a	N	Y	Y	Y	Y	Y	Y	Y	Y	Y	Y	Y	Y	Y	Y	Y	Y	Y	94.44%
Items16b	N	Y	Y	Y	Y	Y	Y	Y	Y	Y	Y	Y	Y	Y	N	Y	Y	Y	88.89%
Items17	N	Y	Y	Y	Y	Y	Y	Y	Y	Y	Y	Y	Y	Y	Y	N	Y	Y	88.89%
Items18	N	N	N	N	Y	Y	N	Y	Y	Y	Y	Y	N	Y	N	Y	Y	Y	61.11%
Items19	Y	Y	Y	Y	Y	Y	Y	Y	Y	Y	Y	Y	Y	Y	Y	Y	Y	Y	100%
Items20a	Y	Y	PY	Y	Y	Y	N	N	Y	Y	Y	Y	N	Y	N	N	Y	Y	72.22%
Items20b	Y	Y	Y	Y	Y	Y	N	Y	Y	Y	Y	Y	Y	Y	N	N	Y	Y	83.33%
Items20c	Y	Y	Y	N	N	Y	N	N	N	N	N	Y	Y	Y	N	Y	N	Y	50%
Items20d	N	N	Y	Y	Y	Y	N	N	Y	Y	N	Y	Y	N	N	N	Y	N	50%
Items21	N	N	Y	Y	Y	Y	N	N	N	N	Y	Y	N	Y	Y	N	Y	Y	55.56%
Items22	N	N	N	Y	N	N	N	N	N	N	Y	N	Y	N	N	N	N	N	16.67%
Items23a	Y	Y	Y	Y	Y	Y	Y	Y	Y	Y	Y	Y	Y	Y	N	N	Y	N	83.33%
Items23b	Y	Y	Y	Y	Y	Y	Y	Y	Y	Y	Y	Y	Y	Y	N	N	Y	Y	88.89%
Items23c	Y	Y	Y	Y	N	Y	Y	Y	Y	Y	Y	Y	Y	Y	N	N	Y	Y	83.33%
Items23d	Y	Y	Y	Y	Y	Y	Y	Y	Y	Y	Y	Y	Y	Y	Y	N	Y	Y	94.44%
Items24a	Y	Y	N	N	N	N	N	N	N	Y	N	N	N	N	N	N	N	N	16.67%
Items24b	Y	Y	N	N	N	N	N	N	N	Y	N	N	N	N	N	N	N	N	16.67%
Items24c	N	N	N	N	N	N	N	N	N	N	N	N	N	N	N	N	N	N	0%
Items25	Y	Y	N	Y	Y	Y	Y	N	Y	Y	N	N	N	N	N	N	N	N	44.44%
Items26	Y	Y	N	Y	Y	Y	Y	N	N	Y	N	N	Y	N	N	N	N	N	44.44%
Items27	Y	Y	N	Y	N	Y	N	N	Y	Y	N	N	N	N	N	N	N	N	33.33%

*Y* yes, *N* no, *PY* partially yes

The “a, b, c, d, e, f” represent sub-items

Further statistical analysis indicated disparities between “fully compliant” and “not compliant” items in English and Chinese literature. The percentage of “fully compliant” items was 54.76% for English literature versus 50.45% for Chinese literature, with a mean of 23 and 21.125 for Chinese literature. Regarding the number of “non-compliant” items, the percentage for English literature was 35.24%, while for Chinese literature, it was 38.51%; the mean number was 14.8 for English versus 16.125 for Chinese literature. The difference between partially compliant items was minimal. Compared to Chinese literature, English literature had better full compliance and non-compliance. Table 4 shows the reported status and percentage for English literature, while Table 5 shows the same for Chinese literature.

**Table 4:** The reporting situation and proportion in English literature

Authors, year	Number of fully conforming entries (percent of rate)	Number of partially conforming entries (percent of rate)	Number of non-conforming entries (percent of rate)
George A Kelley 2022 [22]	24(57.14%)	2(4.76%)	16(38.1%)
Jiale Guo 2022 [23]	8(19.05%)	4(9.52%)	30(71.43%)
Jun-Hong Yan 2013 [24]	24(57.14%)	6(14.29%)	12(28.57%)
Lidong Hu 2021 [25]	27(64.29%)	4(9.52%)	11(26.19%)
R. Lauche 2013 [26]	27(64.29%)	5(11.90%)	10(23.81%)
Ruojin Li 2020 [27]	30(71.43%)	5(11.90%)	7(16.67%)
Wen-Dien Chang 2016 [28]	16(38.10%)	2(4.76%)	24(57.14%)
Yanwei You 2021 [29]	18(42.86%)	5(11.90%)	19(45.24%)
Yingjie Zhang 2017 [30]	25(59.52%)	5(11.90%)	12(28.57%)
Zhi-peng Zeng 2020 [31]	31(73.81%)	4(9.52%)	7(16.67%)
Mean	23	4.2	14.8
Total percentage	54.76%	10%	35.24%

**Table 5:** The reporting situation and proportion in Chinese literature

Authors, year	Number of fully conforming entries (% of rate)	Number of partially conforming entries (% of rate)	Number of non-conforming entries (% of rate)
ZENG Ling-feng 2018 [32]	25(59.52%)	5(11.90%)	12(28.57%)
Li Ruojin 2021 [33]	25(59.52%)	5(11.90%)	12(28.57%)
Li Zimeng 2020 [34]	24(57.14%)	4(9.52%)	13(30.95%)
WANG Li-dong 2022 [35]	25(59.52%)	5(11.90%)	12(28.57%)
LIU Wen-jun 2020 [36]	10(23.81%)	5(11.90%)	27(64.29%)
XIA Lu-qin 2020 [37]	11(26.19%)	3(7.14%)	28(66.67%)
XIE Hui 2016 [38]	26(61.90%)	5(11.90%)	11(26.19%)
XIE Yu 2015 [39]	23(54.76%)	5(11.90%)	14(33.33%)
Mean	21.125	4.625	16.125
Total percentage	50.45%	11.04%	38.51%

#### The risk of bias of the included SRs/MAs

The risk of bias was assessed using the ROBIS tool, which consists of four risk of bias domains (phase 2), three summary landmark questions (phase 3), and a final risk of bias judgment. 11(11/18, 61.11%) of the 18 included studies were assessed to be at high risk. 1(1/18, 5.56%) study was at high risk for “Inclusion Criteria (Domain 1)”. 10(10/18, 55.56%) studies were at high risk for “Retrieval and Screening (Domain 2)”. Eight (8/18, 44.44%) studies’ “Data Extraction (Domain 3)” failed to provide sufficient judgmental information designated as unclear risk. 6(8/18, 44.44%) studies’ “Data Extraction (Domain 3)” was high risk. 16(16/18, 88.89%) studies had “Data Processing (Domain 4)” as high risk. 12(12/18, 66.67%) studies had none of the risks associated with phase 2 explained and addressed. Thus, phase 3 of Q1 was “No,” 2(2/18, 11.11%) studies did not reasonably consider the included studies' relevance to systematically evaluating research questions. Thus, Q2 was “No”. 18(18/18, 100.00%) studies avoided overemphasizing statistically different results. Thus, Q3 was “Yes.” For details, see Table 6. To summarize, the main reasons for the high risk of bias were (i) failure to search the trial registry, no risk of bias detection, and possible reporting bias; (ii) failure to deal with inter-study heterogeneity; (iii) the stability of the results was unknown; and (iv) failure to elucidate the above limitations in the discussion section. Overall, the risk of bias of the nine SRs included was high, which may affect the results, and it is necessary to standardize the study methods to reduce the risk of bias.

**Table 6:** Risk of bias for the included SRs/MAs

Authors, year	Phase 2				Phase 3			Overall quality
	Domain 1	Domain 2	Domain 3	Domain 4	Q1	Q2	Q3	
George A Kelley 2022 [22]	√	x	x	x	N	Y	Y	High
Jiale Guo 2022 [23]	√	x	√	x	N	Y	Y	High
Jun-Hong Yan 2013 [24]	√	√	?	x	N	Y	Y	High
Lidong Hu 2021 [25]	√	√	√	x	Y	Y	Y	Low
R. Lauche 2013 [26]	√	√	√	x	N	Y	Y	High
Ruojin Li 2020 [27]	√	x	?	x	Y	Y	Y	Low
Wen-Dien Chang 2016 [28]	x	x	x	x	N	Y	Y	High
Yanwei You 2021 [29]	√	x	?	x	Y	Y	Y	Low
Yingjie Zhang 2017 [30]	√	√	√	x	N	Y	Y	High
Zhi-peng Zeng 2020 [31]	√	√	?	x	Y	Y	Y	Low
ZENG Ling-feng 2018 [32]	√	√	x	x	N	Y	Y	High
Li Ruojin 2021 [33]	√	x	x	√	N	Y	Y	High
Li Zimeng 2020 [34]	√	x	?	x	N	Y	Y	High
WANG Li-dong 2022 [35]	√	x	?	√	Y	Y	Y	Low
LIU Wen-jun 2020 [36]	√	x	x	x	N	N	Y	High
XIA Lu-qin 2020 [37]	√	x	x	x	N	N	Y	High
XIE Hui 2016 [38]	√	√	?	x	N	Y	Y	Low
XIE Yu 2015 [39]	√	√	?	x	Y	Y	Y	Low

“√” = low risk; “x” = high risk; “?” = unclear risk. *Domain 1* study eligibility criteria, *Domain 2* identification and selection of studies; *Domain 3* data collection and study appraisal; *Domain 4* synthesis and findings. *Q1* whether all risks of bias in phase 2 were addressed or explained, *Q2* whether correlations between the original study and the meta were considered, *Q3* Whether to avoid emphasizing only statistically significant results: “N” for “No,” “Y” for “Yes”

#### Evidence quality of the included SRs/MAs

A total of 93 pieces of evidence were extracted, of which 46 (49.46%, 46/93) were very low quality, 34 (36.56%, 34/93) were low quality, 13 (13.98%, 13/93) were moderate quality, and there was no high-quality evidence. 100% (93/93) of the evidence was downgraded due to limitations; 54.84% (51/93) was downgraded due to substantial heterogeneity (I2 50%); 68.82% (64/93) was downgraded due to imprecision; and 13.98% (13/93) was downgraded due to publication bias. Seventy-six pieces of evidence (81.72%, 76/93) showed TCE was more effective than control, while 17 (18.28%, 76/93) revealed no statistically significant difference between the two groups. Table 7 outlines the GRADE downgrading process.

**Table 7:** The assessment of GRADE

Author, publication year	Intervention measures (Intervention vs. Control)	Outcomes (Studies, sample size)	Relative effect (95%CI)	*P* value	*I*^2^	Limitations	Inconsistency	Indirectness	Imprecision	Publication bias	Quality
George A Kelley 2022 [22]	Tai Chi vs. Control group	WOMAC pain (8,407)	ES = − 0.75, 95%CI (− 0.99, − 0.51)	*P* = 0.26	*I*^2^ = 21%	− 1	0	0	0	0	⨁⨁⨁◯1
		WOMAC stiffness (8,407)	ES = − 0.7, 95%CI (− 0.95, − 0.46)	*P* = 0.21	*I*^2^ = 27%	− 1	0	0	0	0	⨁⨁⨁◯^1^
		WOMAC physical function (8,407)	ES = − 0.91, 95%CI (− 1.12, − 0.7)	*P* = 0.40	*I*^2^ = 3%	− 1	0	0	0	0	⨁⨁⨁◯^1^
Jiale Guo 2022 [23]	Wuqinxi vs. Control group	WOMAC (6,560)	MD = − 105.76, 95%CI (− 161.38, − 50.14)	*P* < 0.01	*I*^2^ = 85%	− 1	− 1	0	0	0	⨁⨁◯◯^1,2^
		Pain (4,414)	MD = − 17.00, 95%CI (− 21.41, − 12.58)	*P* < 0.00001	*I*^2^ = 0%	− 1	0	0	0	0	⨁⨁⨁◯^1^
		Stiffness (4,414)	MD = − 3.43, 95%CI (− 5.50, − 1.37)	*P* = 0.001	*I*^2^ = 0%	− 1	0	0	0	0	⨁⨁⨁◯^1^
		Physical function (4,414)	MD = − 33.45, 95%CI (− 48.74, − 18.17)	*P* < 0.0001	*I*^2^ = 0%	− 1	0	0	0	0	⨁⨁⨁◯^1^
		VAS (2,136)	MD = − 1.07, 95%CI (− 1.97, − 0.17)	*P* = 0.02	*I*^2^ = 69%	− 1	− 1	0	− 1	0	⨁◯◯◯^1,2,3^
Jun-Hong Yan 2013 [24]	Tai Chi vs. Control group	WOMAC pain (7,348)	SMD = − 0.45, 95%CI (− 0.70, − 0.20)	*P* = 0.0005	*I*^2^ = 21%	− 1	0	0	− 1	− 1	⨁◯◯◯^1,3,4^
		WOMAC stiffness (5,237)	SMD = − 0.31, 95%CI (− 0.60, − 0.02)	*P* = 0.04	*I*^2^ = 17%	− 1	0	0	− 1	− 1	⨁◯◯◯^1,3,4^
		WOMAC physical function (5,219)	SMD = − 0.61, 95%CI (− 0.85, − 0.37)	*P* < 0.00001	*I*^2^ = 0%	− 1	0	0	− 1	− 1	⨁◯◯◯^1,3,4^
Lidong Hu 2021 [25]	Tai Chi vs. No exercise	WOMAC pain (14, 877)	SMD = − 0.69, 95%CI (− 0.95, − 0.44)	*P* < 0.001	*I*^2^ = 67%	− 1	− 1	0	0	0	⨁⨁◯◯^1,2^
		WOMAC stiffness (12, 769)	SMD = − 0.65, 95%CI (− 0.98, − 0.33)	*P* < 0.01	*I*^2^ = 77%	− 1	− 1	0	0	0	⨁⨁◯◯^1,2^
		WOMAC physical function (13, 844)	SMD = − 0.92, 95%CI (− 1.16, − 0.69)	*P* < 0.001	*I*^2^ = 57%	− 1	− 1	0	0	0	⨁⨁◯◯^1,2^
		6MWT (6,426)	SMD = 0.55, 95%CI (0.10, 0.99)	*P* = 0.02	*I*^2^ = 74%	− 1	− 1	0	0	0	⨁⨁◯◯^1,2^
		TUG (5,225)	SMD = − 0.55, 95%CI (− 0.82, − 0.29)	*P* < 0.001	*I*^2^ = 0%	− 1	0	0	− 1	0	⨁⨁◯◯^1,3^
		Balance score (4,175)	SMD = 0.69, 95%CI (0.38, 0.99)	*P* < 0.001	*I*^2^ = 39%	− 1	0	0	− 1	0	⨁⨁◯◯^1,3^
		SF-36PCS (5,409)	SMD = 0.48, 95%CI (0.28, 0.68)	*P* < 0.001	*I*^2^ = 0.0191%	− 1	0	0	0	0	⨁⨁⨁◯^1^
		SF-36CS (5,409)	SMD = 0.26, 95%CI (0.06,0.45)	*P* = 0.01	*I*^2^ = 0%	− 1	0	0	0	0	⨁⨁⨁◯^1^
		Depression score (3,319)	SMD = − 0.46, 95%CI (− 0.68, − 0.24)	*P* < 0.001	*I*^2^ = 3%	− 1	0	0	− 1	0	⨁⨁◯◯^1,3^
		ASES (4,352)	SMD = 0.27, 95%CI (0.06,0.48)	*P* = 0.01	*I*^2^ = 44%	− 1	0	0	− 1	0	⨁⨁◯◯^1,3^
R. Lauche 2013 [26]	Tai Chi vs Control group	WOMAC/VAS pain (5,215)	SMD = − 0.72, 95%CI (− 1.00, − 0.44)	*P* < 0.00001	*I*^2^ = 0%	− 1	0	0	− 1	0	⨁⨁◯◯^1,3^
		WOMAC physical function (5,215)	SMD = − 0.72, 95%CI (− 1.01, − 0.44)	*P* < 0.00001	*I*^2^ = 0%	− 1	0	0	− 1	0	⨁⨁◯◯^1,3^
		WOMAC stiffness (5,215)	SMD = − 0.59, 95%CI (− 0.99, − 0.19)	*P* = 0.004	*I*^2^ = 50%	− 1	− 1	0	− 1	0	⨁◯◯◯^1,2,3^
		SF-36 MCS. (2,84)	SMD = 0.35, 95%CI (− 0.31,1.01)	*P* = 0.29	*I*^2^ = 54%	− 1	− 1	0	− 1	0	⨁◯◯◯^1,2,3^
		SF-36 PCS (2,84)	SMD = 0.88, 95%CI (0.42,1.34)	*P* = 0.0002	*I*^2^ = 0%	− 1	0	0	− 1	0	⨁⨁◯◯^1,3^
Ruojin Li 2020 [27]	TCE vs. Control group	WOMAC/KOOS pain (15,839)	SMD = − 0.61, 95%CI (− 0.86, − 0.37)	*P* < 0.00001	*I*^2 ^= 64%	− 1	− 1	0	0	− 1	⨁◯◯◯^1,2,4^
		WOMAC/KOOS stiffness (14,732)	SMD = − 0.75, 95%CI (− 1.09, − 0.41)	*P* < 0.0001	*I*^2^ = 77%	− 1	− 1	0	0	− 1	⨁◯◯◯^1,2,4^
		WOMAC/KOOS physical function (14,796)	SMD = − 0.67, 95%CI (− 0.82, − 0.53)	*P* < 0.00001	*I*^2^ = 34%	− 1	0	0	0	− 1	⨁⨁◯◯^1,4^
Wen-Dien Chang 2016 [28]	Tai Chi vs. Control group	WOMAC pain (6,250)	SMD = − 0.41, 95%CI (− 0.67, − 0.14)	*P* = 0.0001	*I*^2^ = 80%	− 1	− 1	0	− 1	0	⨁◯◯◯^1,2,3^
		WOMAC stiffness (6,250)	SMD = − 0.20, 95%CI (− 0.45, − 0.05)	*P* = 0.02	*I*^2^ = 59%	− 1	− 1	0	− 1	0	⨁◯◯◯^1,2,3^
		WOMAC physical function (5,207)	SMD = − 0.16, 95%CI (− 0.44, − 0.11)	*P* = 0.14	*I*^2^ = 41%	− 1	0	0	− 1	0	⨁⨁◯◯^1,3^
		6MWT (3,97)	SMD = − 0.16, 95%CI (− 1.23,0.90)	*P* = 0.003	*I*^2^ = 82%	− 1	− 1	0	− 1	0	⨁◯◯◯^1,2,3^
		SAFE (2,134)	SMD = − 0.63, 95%CI (− 0.98, − 0.27)	*P* = 0.78	*I*^2^ = 99%	− 1	− 1	0	− 1	0	⨁◯◯◯^1,2,3^
		Stair climb test (2,53)	SMD = − 0.74, 95%CI (− 1.34, − 0.15)	*P* = 0.04	*I*^2^ = 74%	− 1	− 1	0	− 1	0	⨁◯◯◯^1,2,3^
Yanwei You 2021 [29]	Tai Chi vs Control group	6MWT (5,273)	MD = 46.67, 95%CI (36.91,56.43)	*P* < 0.00001	*I*^2^ = 1%	− 1	0	0	− 1	0	⨁⨁◯◯^1,3^
		TUG (6,306)	MD = − 0.89, 95%CI (− 1.16, − 0.61)	*P* < 0.00001	*I*^2^ = 16%	− 1	0	0	− 1	0	⨁⨁◯◯^1,3^
		WOMAC physical function (8,443)	MD = − 11.28, 95%CI (− 13.33, − 9.24)	*P* < 0.00001	*I*^2^ = 0%	− 1	0	0	0	0	⨁⨁⨁◯^1^
Yingjie Zhang 2017 [30]	TCE vs. Control group	Pain (8,325)	SMD = − 0.77, 95%CI (− 1.13, − 0.41)	*P* < 0.0001	*I*^2^ = 54%	− 1	− 1	0	− 1	0	⨁◯◯◯^1,2,3^
		Physical function (8,325)	SMD = − 0.75, 95%CI (− 0.98, − 0.52)	*P* < 0.00001	*I*^2^ = 37%	− 1	0	0	− 1	0	⨁⨁◯◯^1,3^
		Stiffness (7,228)	SMD = − 0.56, 95%CI (− 0.96, − 0.16)	*P* = 0.006	*I*^2^ = 51%	− 1	− 1	0	− 1	0	⨁◯◯◯^1,2,3^
		SF-36 PCS (3,105)	SMD = 0.57, 95%CI (0.17,0.97)	*P* = 0.005	*I*^2^ = 0%	− 1	0	0	− 1	0	⨁⨁◯◯^1,3^
		SF-36 MCS (3,105)	SMD = 4.12, 95%CI (− 0.50,8.73)	*P* = 0.08	*I*^2^ = 52%	− 1	− 1	0	− 1	0	⨁◯◯◯^1,2,3^
Zhi-peng Zeng 2020 [31]	Baduanjin vs. waiting list control	WOMAC pain (3,186)	MD = − 4.40, 95%CI (− 7.16, − 1.64)	*P* = 0.002	*I*^2^ = 96%	− 1	− 1	0	− 1	0	⨁◯◯◯^1,2,3^
		WOMAC stiffness (3,186)	MD = − 1.34, 95%CI (− 1.64, − 1.04)	*P* < 0.00001	*I*^2^ = 57%	− 1	− 1	0	− 1	0	⨁◯◯◯^1,2,3^
		WOMAC physical function (3,186)	MD = − 2.44, 95%CI (− 4.33, − 0.55)	*P* = 0.01	*I*^2^ = 85%	− 1	− 1	0	− 1	0	⨁◯◯◯^1,2,3^
	Baduanjin VShealth education	WOMAC pain (2,116)	MD = − 1.69, 95%CI (− 2.03, − 1.35)	*P* < 0.00001	*I*^2^ = 0%	− 1	0	0	− 1	0	⨁⨁◯◯^1,3^
		WOMAC stiffness (2,116)	MD = − 0.86, 95%CI (− 1.13, − 0.58)	*P* < 0.00001	*I*^2^ = 0%	− 1	0	0	− 1	0	⨁⨁◯◯^1,3^
		WOMAC physical function (2,116)	MD = − 2.23, 95%CI (− 3.65, − 0.82)	*P* = 0.002	*I*^2^ = 0%	− 1	0	0	− 1	0	⨁⨁◯◯^1,3^
ZENG Ling-feng 2018 [32]	TCE vs. Control group	WOMAC pain-short term (8,412)	SMD = − 1.40, 95%CI (− 2.28, − 0.52)	*P* = 0.002	*I*^2^ = 93%	− 1	− 1	0	0	− 1	⨁◯◯◯^1,2,4^
		WOMAC pain-long term (2,73)	SMD = − 0.29, 95%CI (− 1.06, − 0.48)	*P* = 0.46	*I*^2^ = 62%	− 1	− 1	0	− 1	0	⨁◯◯◯^1,2,3^
		WOMAC stiffness-short term (7,288)	SMD = − 0.48, 95%CI (− 0.87, − 0.08)	*P* = 0.02	*I*^2^ = 61%	− 1	− 1	0	− 1	− 1	⨁◯◯◯^1,2,3^
		WOMAC stiffness-long term (2,71)	SMD = 0.06, 95%CI (− 0.72,0.83)	*P* = 0.89	*I*^2^ = 62%	− 1	− 1	0	− 1	0	⨁◯◯◯^1,2,3^
		WOMAC physical function-short term (8,412)	SMD = − 1.92, 95%CI (− 3.16, − 0.68)	*P* = 0.002	*I*^2^ = 96%	− 1	− 1	0	0	− 1	⨁◯◯◯^1,2,4^
		WOMAC physical function-long term (2,71)	SMD = − 0.35, 95%CI (− 0.82, − 0.13)	*P* = 0.15	*I*^2^ = 40%	− 1	0	0	− 1	− 1	⨁◯◯◯^1,3,4^
		SF-36-short term (2,84)	SMD = 0.89, 95%CI (0.43, 1.35)	*P* = 0.0002	*I*^2^ = 0%	− 1	0	0	-1	-1	⨁◯◯◯^1,3,4^
		SF-36-long term (2,84)	SMD = 4.82, 95%CI (− 4.85, 14.49)	*P* = 0.33	*I*^2^ = 74%	− 1	− 1	0	− 1	0	⨁◯◯◯^1,2,3^
Li Ruojin 2021 [33]	TCE vs. Control group	WOMAC/KOOS pain (28,1800)	SMD = − 0.50, 95%CI (− 0.67, − 0.33)	*P* < 0.00001	*I*^2^ = 67%	− 1	− 1	0	0	− 1	⨁◯◯◯^1,2,4^
		WOMAC/KOOS stiffness (26,1635)	SMD = − 0.59, 95%CI (− 0.82, − 0.37)	*P* < 0.00001	*I*^2^ = 79%	− 1	− 1	0	0	− 1	⨁◯◯◯^1,2,4^
		WOMAC/KOOS physical function (28,1800)	SMD = − 0.71, 95%CI (− 0.89, − 0.53)	*P* < 0.00001	*I*^2^ = 71%	− 1	− 1	0	0	0	⨁⨁◯◯^1,2^
Li Zimeng 2020 [34]	Baduanjin.vs Control group	VAS/WOMAC pain (6,297)	SMD = − 1.50, 95%CI (− 2.43, − 0.58)	*P* = 0.001	*I*^2^ = 90%	− 1	− 1	0	− 1	0	⨁◯◯◯^1,2,3^
		Stiffness (4,170)	SMD = − 0.85, 95%CI (− 1.46, − 0.23)	*P* = 0.007	*I*^2^ = 70%	− 1	− 1	0	− 1	0	⨁◯◯◯^1,2,3^
		Physical function (4,170)	SMD = − 1.28, 95%CI (− 2.45, − 0.10)	*P* = 0.03	*I*^2^ = 91%	− 1	− 1	0	− 1	0	⨁◯◯◯^1,2,3^
		6MWT (2,66)	SMD = 1.04, 95%CI (0.07, 2.02)	*P* = 0.03	*I*^2^ = 68%	− 1	− 1	0	− 1	0	⨁◯◯◯^1,2,3^
WANG Li-dong 2022 [35]		WOMAC/KOOS pain (13,852)	SMD = − 0.84, 95%CI (− 1.10, − 0.58)	*P* < 0.05	*I*^2^ = 63.6%	− 1	− 1	0	0	0	⨁⨁◯◯^1,2,^
		Stiffness (13,852)	SMD = − 0.79, 95%CI (− 1.09, − 0.48)	*P* < 0.05	*I*^2^ = 73.5%	− 1	− 1	0	0	0	⨁⨁◯◯^1,2,^
		Physical function (13,852)	SMD = − 0.88, 95%CI (− 1.19, − 0.57)	*P* < 0.05	*I*^2^ = 74.1%	− 1	− 1	0	0	0	⨁⨁◯◯^1,2,^
		TUG (5,246)	SMD = 0.6, 95%CI (0.11, 1.09)	*P* < 0.05	*I*^2^ = 76.0%	− 1	− 1	0	− 1	0	⨁◯◯◯^1,2,3^
		6 MWT(6,426)	SMD = − 0.65, 95%CI (− 0.91, − 0.38)	*P* < 0.05	*I*^2^ = 0%	− 1	0	0	0	0	⨁⨁⨁◯^1^
LIU Wen-jun 2020 [36]	Tai Chi vs. Control group	WOMAC pain (9,499)	SMD = − 0.78, 95%CI (− 0.97, − 0.59)	*P* < 0.00001	*I*^2^ = 43%	− 1	0	0	0	0	⨁⨁⨁◯^1^
		WOMAC stiffness (6,299)	SMD = − 0.78, 95%CI (− 1.24, − 0.32)	*P* = 0.0008	*I*^2^ = 71%	− 1	− 1	0	− 1	0	⨁◯◯◯^1,2,3^
		WOMAC physical function (7,371)	SMD = − 0.80, 95%CI (− 1.01, − 0.58)	*P* < 0.00001	*I*^2 ^= 34%	− 1	0	0	− 1	0	⨁⨁◯◯^1,3^
		TUG (6,294)	SMD = − 0.56, 95%CI (− 1.00, − 0.12)	*P* = 0.01	*I*^2^ = 69%	− 1	− 1	0	− 1	0	⨁◯◯◯^1,2,3^
		6MWT (4,152)	SMD = 0.82, 95%CI (0.21, 1.44)	*P* = 0.009	*I*^2^ = 68%	− 1	− 1	0	− 1	0	⨁◯◯◯^1,2,3^
XIA Lu-qin 2020 [37]	Tai Chi vs. Control group	WOMAC (8,374)	SMD = − 0.57, 95%CI (− 0.98, − 0.17)	*P* = 0.005	*I*^2^ = 70%	− 1	− 1	0	− 1	0	⨁◯◯◯^1,2,3^
XIE Hui 2016 [38]	Tai Chi vs. Control group	Pain (11,477)	SMD = − 0.64, 95%CI (− 0.90, − 0.38)	*P* < 0.00001	*I*^2^ = 42%	− 1	0	0	0	0	⨁⨁⨁◯^1^
		Physical function (10,465)	SMD = − 0.78, 95%CI (− 1.01, − 0.55)	*P* < 0.00001	*I*^2 ^= 29%	− 1	0	0	0	0	⨁⨁⨁◯^1^
		Stiffness (7,276)	SMD = − 0.89, 95%CI (− 1.43, − 0.35)	*P* = 0.001	*I*^2^ = 77%	− 1	− 1	0	− 1	0	⨁◯◯◯^1,2,3^
		TUG (4,235)	MD = − 1.61, 95%CI (− 3.18, − 0.04)	*P* = 0.04	*I*^2^ = 90%	− 1	− 1	0	− 1	0	⨁◯◯◯^1,2,3^
		STS (3,128)	MD = − 3.28, 95%CI (− 6.34, − 0.22)	*P* = 0.04	*I*^2^ = 87%	− 1	− 1	0	− 1	0	⨁◯◯◯^1,2,3^
		6MWT (7,326)	SMD = 0.71, 95%CI (0.24, 1.19)	*P* = 0.003	*I*^2^ = 74%	− 1	− 1	0	− 1	0	⨁◯◯◯^1,2,3^
		Balance score (5,195)	SMD = 0.68, 95%CI (0.33, 1.102)	*P* = 0.0001	*I*^2^ = 25%	− 1	0	0	− 1	0	⨁⨁◯◯^1,3^
		SF-12/36 (3,181)	SMD = 0.44, 95%CI (− 0.03, 0.90)	*P* = 0.07	*I*^2^ = 53%	− 1	− 1	0	− 1	0	⨁◯◯◯^1,2,3^
XIE Yu 2015 [39]	Tai Chi vs. Control group	Pain (6,251)	SMD = − 0.73, 95%CI (− 0.99, − 0.47)	*P* < 0.00001	*I*^2^ = 39%	− 1	0	0	− 1	0	⨁⨁◯◯^1,3^
		Physical function (6,249)	SMD = − 0.76, 95%CI (− 1.02, − 0.50)	*P* < 0.00001	*I*^2^ = 0%	− 1	0	0	− 1	0	⨁⨁◯◯^1,3^
		Stiffness (6,249)	SMD = − 0.72, 95%CI (− 1.24, − 0.20)	*P* = 0.007	*I*^2^ = 74%	− 1	− 1	0	− 1	0	⨁◯◯◯^1,2,3^
		Balance score (3,116)	SMD = 0.42, 95%CI (− 0.15, 1.00)	*P* = 0.15	*I*^2^ = 58%	− 1	− 1	0	− 1	0	⨁◯◯◯^1,2,3^
		SF-36 MCS (2,84)	SMD = 0.39, 95%CI (− 0.35, 1.14)	*P* = 0.30	*I*^2^ = 64%	− 1	− 1	0	− 1	0	⨁◯◯◯^1,2,3^
		SF-36 PCS (2,84)	SMD = 0.71, 95%CI (0.25, 1.16)	*P* = 0.002	*I*^2^ = 18%	− 1	0	0	− 1	0	⨁⨁◯◯^1,3^
		6MWT (2,82)	SMD = 0.57, 95%CI (0.11, 1.02)	*P* = 0.01	*I*^2^ = 0%	− 1	0	0	− 1	0	⨁⨁◯◯^1,3^
		BMI (2,83)	MD = − 0.13, 95%CI (− 0.46, 0.21)	*P* = 0.46	*I*^2^ = 0%	− 1	0	0	− 1	0	⨁⨁◯◯^1,3^
		Muscle strength (extensor) (2,99)	SMD = 0.23, 95%CI (− 0.17, 0.63)	*P* = 0.26	*I*^2^ = 35%	− 1	0	0	− 1	0	⨁⨁◯◯^1,3^
		Muscle strength (flexor) (2,99)	SMD = 0.40, 95%CI (− 0.46, 01.25)	*P* = 0.36	*I*^2^ = 75%	− 1	− 1	0	− 1	0	⨁◯◯◯^1,2,3^

*1* downgrade 1 level, *0* no downgrade. ⨁◯◯◯: very low quality; ⨁⨁◯◯: low quality; ⨁⨁⨁◯: medium quality; ⨁⨁⨁⨁: high quality. Reason for downgrade: *1* The included studies have a large bias in methodologies such as randomization, allocation concealment, and blinding. *2* The confidence interval overlaps less, or the *I*^2^ value of the combined results is larger. *3* The sample size from the included studies does not meet the optimal sample size, or the 95% confidence interval crosses the invalid line. *4* The funnel chart is asymmetric

*SMD* the standardized mean difference effect size, *MD* mean difference, *CI* confidence interval

### Security of the included SRs/MAs

None of the SRs/MAs quantified the adverse effects of TCE on knee osteoarthritis. However, six articles [26, 31, 32, 34, 38, 39] indicated the safety of TCE for knee osteoarthritis. Therefore, the safety profile of TCE for knee osteoarthritis appears favorable.

## Discussion

### Mechanism of TCE in KOA treatment

In recent years, more attention has been paid to knee osteoarthritis due to population aging. Guidelines for TCM treatment of knee osteoarthritis [1, 2] recommend TCE for knee osteoarthritis. The 2019 American College of Rheumatology (ACR) guidelines [40] strongly suggest Taiji for knee osteoarthritis, indicating the worldwide use of Taiji exercises. As a physical exercise, Taiji exercises have proven benefits for chronic joint conditions, especially in older adults. Yijinjing originates from an ancient Chinese health-cultivating approach, and research shows it significantly improves knee flexion and relieves joint pain [41]. Yijinjing integrates Traditional Chinese theory with fitness walking, which can enhance the coordination and balance between the internal and external environment of the body, build muscle strength, improve muscle flexibility and endurance, and reduce ligament strains. Baduanjin [42] and Wuqinxi [43] are also performed with precise movement postures to promote blood circulation in joint areas, smooth the passage of qi and blood through meridians, soothe pain, and increase lower limb flexibility and suppleness, thus reducing pain and dysfunction in knee osteoarthritis patients. This study aims to examine the efficacy and influence of TCE on rehabilitating knee osteoarthritis patients of different ages, providing a scientific basis for developing tailored therapies. Patients may exercise less if isolated, especially after the onset of coronavirus disease (COVID). As a physical and mental exercise, TCE has distinct benefits and can be practiced at home during COVID-19. The findings indicate TCE improves functional impairment, pain, psychological status, quality of life, and other conditions in knee osteoarthritis patients. However, due to the poor quality of the studies, the data quality needs improvement and it cannot be concluded that TCE is superior to the control group or other treatments.

### Summary of main results

This is the first review of SRs/MAs on the effectiveness and safety of TCE for knee osteoarthritis. Using AMSTAR 2, PRISMA2020, and GRADE, the published SRs and MAs were assessed. Additionally, over 70% of all 11 [18–22, 25–28, 30, 33] SRs/MAs were adequately reported according to the PRISMA2020 checklist. However, the evidence quality of graded outcomes could have been better. Systematic, high-quality reviews can produce less biased, more scientific evidence for clinical practice and health decisions [44].

All SRs/MAs examined by MSTAR 2 had at least one critical flaw, and the methodological quality of the 18 included publications was deemed “very low.” The primary methodological quality issues were:

- The lack of a pre-defined review protocol compromised the rigor of the systematic review.
- No excluded studies were identified, which did not facilitate assessing clinical heterogeneity.
- Lack of proper referencing, trial registry establishment, and further investigation of gray literature.
- There is no assessment of the overall effect of risk of bias in RCTs.
- There is no investigation of potential sources of heterogeneity to help interpret meta-analysis results.

Among the 18 included papers, the top three items with the most “non-conformities” in PRISMA 2020 were item 13b (100%), item 24c (100%), item 13c (83.33%), item 15 (83.33%), item 22 (83.33%), item 24a (83.33%), item 24c (83.33%), item 24a (83.33%), and item 24b (83.33%). Therefore, it can be determined that the primary reporting flaws are the following:

- Lack of description of preprocessing before data merging (missing data, transformation).
- Definition and explanation of discrepancies from registration information.
- Description of presentation method of findings graphs or tables.
- Description of the method to assess the quality of evidence for each outcome (e.g., using GRADE).
- Presentation of thresholds.

Differences in journal type, publication dates, and authorship levels can also lead to disparities in reporting standards among works by the same researcher. Regarding defining data extraction methods, interpreting findings in light of other evidence, stating funding sources, and other reporting aspects, the English literature was much more prescriptive than the Chinese literature. All included English journals explicitly referred to PRISMA reporting standards, while none of the Chinese journals did; this may significantly influence the disparate reporting quality between English and Chinese literature.

The top 3 “fully or partially conforming” items out of 18 were: item 3 (94.44%), item 10b (94.44%), item 11 (94.44%), item 16a (94.44%), item 23d (94.44%), item 2 (88.89%), item 5 (88.89%), item 7 (88.89%), item 10a (88.89%), item 16b (88.89%), item 17 (88.89%), and item 23b (88.89%). These items have been part of the PRISMA statement [45] and are becoming increasingly refined as reporting standards evolve.

The 18 studies included in this analysis were published between 2013 and 2021; the more recent the publication date, the better the quality of reporting. The evaluation results revealed the inadequate quality of included studies (only 52.78% (399/756) of “fully conforming” items were 75% [46], indicating the systematic review/meta-analysis on TCM gongfu for knee osteoarthritis lacks normalcy and has room for improvement.

### Recommendations for future research

Due to the low quality of early literature studies, there is debate about whether TCE is more effective for knee osteoarthritis than controls or alternative therapies. Therefore, it is crucial to strengthen methodological quality issues of randomized clinical studies, blinding and allocation concealment in later stages, conduct high quality, extensive sample studies, and multicenter clinical controlled trials. Future evidence-based reviews will also require continued focus on SR/MA approaches and reporting quality to produce high-quality research and provide a proper clinical basis for decision-making.

### Limitations

This study has several limitations: (1) the interventions of included studies, including Taiji, Baduanjin, Wuqinxi, Yijinjing, and Qigong, are complex and heterogeneous, and their effect values cannot be quantitatively combined for analysis; (2) due to database restrictions and subsequent bias, data from included studies may be missing; (3) the low quality of evidence in the original literature and methodological flaws of researchers conducting the systematic reviews may compromise the accuracy of re-evaluation; (4) subjective disputes between researchers over the evaluation process may influence the outcomes and conclusions of the assessment.

## Conclusion

TCE is, therefore, beneficial and safe for knee osteoarthritis. However, clinicians should proceed cautiously from these findings in practice due to the relatively low methodological and evidentiary quality of included SRs/MAs.

### Supplementary Information

Supplementary Material 1. Appendix 1: Retrieval Strategies of Web of Science Databases.

Supplementary Material 2. The 16 items of AMSTAR-2.

Supplementary Material 3. The checklists of PRISMA2020.

## Data Availability

The datasets analyzed during the current study are available from the corresponding author upon reasonable request.
