# Supplementary material for: Evaluation of traditional Chinese exercise for knee osteoarthritis (KOA): an overview of systematic reviews
Source: Syst Rev. 2024 Jul 18;13:187. doi: 10.1186/s13643-024-02606-0 (PMC11256411; doi:10.1186/s13643-024-02606-0)
Supplement: Supplementary file 1 — Supplementary Material 1. Appendix 1: Retrieval Strategies of Web of Science Databases. [file 13643_2024_2606_MOESM1_ESM.docx]

Supplementary Materials

**Appendix 1:Retrieval strategies of Web of Science Databases**

| Query | Search strategies |
| --- | --- |
| 1# | TS=(osteoarthr* OR knee osteoarthritis OR gonarthritis OR KOA OR Knee OA OR Gu-bi disorders OR degenerative arthritis) |
| 2# | TS=(Taiji or "tai chi" or "tai ji" or "tai ji quan" or "tai chi chuan" or Taichiquan or Taijiquan or "t'ai ji" or t'aichi or qigong or Baduanjin or "Baduanjin exercise" or "eight-section brocade" or Yijinjing or "Classic of Changing tendon" or "the classics of tendon changing" or "Yi-Gin-Ching of Bodhidharma" or Wuqinxi or "Five-animal Exercises" or Qigong or "qi gong" or "health qigong" or "Chi Kung" or "traditional Chinese exercise" or "traditional exercise therapy" or "exercise therapy" or "remedial exercise" or "remedial exercises" or "exercise therapies" or "rehabilitation exercise" or "therapeutic Chinese exercises") |
| 3# | #1 AND #2 |
| 4# | TS=("meta analysis" or "meta analyses" or "meta-analysis" or "meta-analyses" or metaanalysis or metanalysis or met-analysis or metaanalyses or metanalyses or met-analyses or "data pooling" or "data poolings" or "clinical trial overview" or "clinical trial overviews" or "systematic review" or "systematic reviews") |
| 5# | #3 AND #4 |
